# Comparable severity of *Plasmodium vivax* and *Plasmodium falciparum* malaria in hospitalized patients in the Peruvian Amazon

**DOI:** 10.1016/j.nmni.2026.101849

**Published:** 2026-09-08

**Authors:** Livia Bresciani, Mariasilvia Guardiani, Manuel Rojas-Blasquez, Edgar Ramirez, Salvatore Iaquinandi, Cesar Martel, Miguel Velarde-Mera, Samar Sifuentes-Vidigal, Anna Carraro, Gianluca Russo, Caterina Pasquazzi, Juan Carlos Celis, Martin Casapia Morales, Emanuele Nicastri, César Ramal Asayag, Miriam Lichtner, Serena Vita

**Affiliations:** a Infectious Diseases Unit, Sapienza of Rome, Rome, Italy; b Department of Public Health and Infectious Diseases, Sapienza University of Rome, Rome, Italy; c Department of Neuroscience, Mental Health and Sense Organs, NESMOS, Sapienza University of Rome, Rome, Italy; d Faculty of Medicine, National University of the Peruvian Amazon, Iquitos, Peru; e Medipeace, Project Assistant, Iquitos, Peru; f Department of Infectious Diseases, Regional Hospital of Loreto, Iquitos, Peru; g National Institute for Infectious Diseases “Lazzaro Spallanzani” IRCCS, Rome, Italy; h Sapienza University of Rome, Sant'Andrea Hospital, Department of Infectious Diseases, Rome, Italy

**Keywords:** Peruvian Amazon, WHO severity criteria, Malaria, *Plasmodium vivax*, Low-transmission setting

## Abstract

**Background:**

*Plasmodium vivax* malaria has long been considered less severe than *P. falciparum* infection. However, there is growing evidence that *P. vivax* can lead to severe and life-threatening disease, although its burden is likely to be underestimated, particularly in low-transmission settings.

This study aimed to contextualize the application of World Health Organization (WHO) severe malaria criteria in a low-transmission setting by describing the clinical and parasitological profile of hospitalized malaria patients in the Peruvian Amazon.

**Methods:**

We conducted a retrospective hospital-based study at Hospital Regional de Loreto, Iquitos, Peru, from January 1, 2023, to October 30, 2025. The total hospital malaria caseload was determined from records assigned a laboratory-confirmed ICD-10 malaria diagnosis. The analytic cohort comprised hospitalized patients with microscopy-confirmed malaria who received intravenous artesunate because of severe malaria according to the WHO 2022 criteria, or inability to tolerate oral antimalarial treatment.

**Results:**

During the study period, 519 laboratory-confirmed malaria diagnoses were recorded in the hospital; of these, 127 patients (24.5%) were hospitalized. Of these, 97 had *P. vivax* infection (76.4%), 27 had *P. falciparum* infection (21.3%), 2 had mixed *P. vivax/P. falciparum* infection (1.6%), and 1 had *P. malariae* infection (0.8%). Overall, 67 patients (52.8%) met criteria for severe malaria, which was observed in 51/97 patients with *P. vivax* (52.6%) and 14/27 patients with *P. falciparum* (51.9%), with no significant difference between species. Notably, patients with *P. vivax* malaria presented with severe manifestations including impaired consciousness, pulmonary oedema or acute respiratory distress syndrome, and shock. Laboratory differences between species were modest, although *P. falciparum* infections were associated with lower haemoglobin and glucose levels, while *P. vivax* was associated with more marked thrombocytopenia. Clinical outcomes were generally favourable, with one death to which malaria-related complications may have contributed.

**Conclusions:**

*P. vivax* contributed substantially to severe malaria in this low-transmission Amazonian setting and showed a severity profile similar to that of *P. falciparum*. The overlap observed between severe and non-severe presentations supports the contextual application of international severity criteria and continued recognition of the clinically important burden of *P. vivax* malaria.

## 1 Introduction

Malaria remains a major public health challenge across tropical regions, with an estimated 282 million cases and 610,000 deaths globally in 2024 [1]. Malaria is clinically classified as either uncomplicated or severe. Severe malaria is defined according to WHO criteria by the presence of one or more clinical or laboratory manifestations of acute organ dysfunction or major metabolic disturbance associated with an increased risk of death [2,3].

Historically, *P. falciparum* has been considered the primary cause of severe malaria, and *P. vivax* has been regarded as relatively benign. This distinction has shaped clinical practice and research priorities [4], largely because *P. falciparum* accounts for most malaria deaths worldwide and most available evidence is from settings where *P. falciparum* predominates [2,5,6]. However, evidence accumulated over the past two decades has challenged this paradigm, showing that *P. vivax* can cause severe, life-threatening complications—including severe anaemia, respiratory distress, acute kidney injury, impaired consciousness, multiorgan dysfunction, and death [2,5,6]. Severe malaria is therefore now recognized as a clinical entity that can occur across *Plasmodium* species. Despite its wide geographic distribution, the manifestations and clinical course of severe *P. vivax* malaria remain incompletely characterized in some endemic regions, particularly in low-transmission settings such as the Peruvian Amazon [7]. In 2024, Peru reported 27,310 *P. vivax* cases, representing 82.0% of the 33,314 malaria cases reported nationally [8]. Given this predominance, even a relatively small proportion of infections progressing to severe disease may represent a substantial clinical burden [9,10].

Although malaria transmission varies considerably within and between endemic regions, published evidence on severe *P. vivax* malaria has been concentrated largely in Asian settings, with comparatively fewer studies from Latin America [11,12]. Differences in transmission intensity, parasite epidemiology, host immunity and healthcare access may limit the direct extrapolation of findings between regions, reinforcing the importance of locally contextualized evidence [7]. In the Peruvian Amazon, malaria transmission is generally low and heterogeneous, potentially limiting the acquisition of naturally acquired immunity [[13], [14], [15]]. In addition, the region's distinct social, cultural and economic context may affect access to diagnosis and treatment and thereby influence disease progression [16]. Although this interaction between context and malaria severity has received considerable research attention over the past decade, characterization of the scale and complexity of the problem remains incomplete [14,16]. Nutritional status and comorbid intestinal parasitic infections may influence prognosis and baseline haematological reserves, potentially affecting malaria severity and recovery [9,16]. The clinical picture may be further complicated by the high burden of other tropical infections, including dengue and other arboviral diseases [10,17].

Vulnerability exists along a spectrum shaped by biological, social and structural factors. Geographic isolation can delay diagnosis and treatment, particularly in riverine and Indigenous communities with limited transport and healthcare infrastructure [16]. Illegal mining adds further vulnerability by altering vector habitats, displacing workers, and creating mobile transmission foci outside formal surveillance. These populations are underrepresented in the clinical literature, as are pregnant women, who face heightened risk of anaemia, recurrent parasitaemia, miscarriage and preterm birth [9,17,18] — compounded by the fact that radical cure cannot be safely given during pregnancy [19].

Finally, malaria epidemiology is not static. Climate and environmental disruption and shifts in human activity alter vector distribution, transmission patterns and population exposure [14], with downstream effects on disease presentation and progression [20]. Local clinical and epidemiological data therefore require periodic updating to reflect current conditions [20].

This study aimed to describe the current demographic, clinical and laboratory characteristics of hospitalized malaria patients in the Peruvian Amazon, compare disease severity and outcomes between *P. vivax* and *P. falciparum*, and contextualize the application of WHO severe malaria criteria.

## 2 Methods

### 2.1 Study design and population

We conducted a retrospective descriptive study at Hospital Regional de Loreto, Iquitos, Peru, from January 1, 2023, to October 30, 2025. To determine the total malaria caseload, we reviewed emergency department and inpatient records for all patients recorded with an ICD-10 malaria code, regardless of whether malaria was the primary reason for presentation or hospitalization: B50 (*Plasmodium falciparum*), B51 (*P. vivax*), B52 (*P. malariae*), B53 (other parasitologically confirmed malaria), or B54 (unspecified malaria). Codes were recorded following laboratory confirmation by microscopy or a malaria rapid diagnostic test.

Patients identified through this hospital-wide review were then assessed for inclusion in the analytic cohort. Hospitalization alone did not determine eligibility, as some patients were admitted for other indications or did not require intravenous antimalarial treatment. The analytic cohort therefore comprised patients with microscopy-confirmed malaria who received at least one dose of intravenous artesunate because of severe malaria or inability to tolerate oral antimalarial treatment, remained hospitalized and survived for at least 24 h, and had laboratory results available at two or more time points. No age restriction was applied.

Severe malaria was defined as the presence of at least one criterion from the 2022 WHO definition: impaired consciousness, prostration, multiple convulsions, acidosis, hypoglycaemia, severe malarial anaemia, renal impairment, jaundice, pulmonary oedema, significant bleeding, or shock criteria [3]. All criteria were assessed except hyperparasitaemia, which could not be evaluated because parasitaemia was reported using a semiquantitative cross-based microscopy system rather than as parasites/μL or percentage parasitaemia. Operational definitions and thresholds are provided in Appendix Table 1.

Data included demographics, clinical data and biochemical data. Recorded risk factors and comorbid conditions were categorized as either WHO-recognized risk factors for severe malaria, including pregnancy and HIV infection, or other comorbidities [3].

### 2.2 Laboratory procedures

Laboratory parameters used to assess severity criteria — haemoglobin, haematocrit, blood glucose, serum creatinine, and serum bilirubin — were measured in all patients at admission (before intravenous artesunate) and at least once thereafter. Additional laboratory parameters routinely measured at the same time points, but not required for severity classification, included mean corpuscular volume (MCV), mean corpuscular haemoglobin (MCH), mean corpuscular haemoglobin concentration (MCHC), white blood cell count, and platelet count.

Malaria was diagnosed by rapid diagnostic testing and/or thick blood-smear microscopy, according to routine clinical practice. Hospitalization was based on clinical assessment and did not require microscopic confirmation. Thick blood-smear microscopy was routinely performed following admission to identify the infecting species, grade parasite density using the WHO cross system [21] and monitor parasitological clearance [21]. Follow-up smears were performed 24, 48 and 72 h after the first artesunate dose, until smear negativity or discharge. Routine laboratory assessments included complete blood count and serum creatinine. Coinfections were defined as additional infectious diseases diagnosed during the index hospitalization. Testing for coinfections was performed when an alternative or additional infectious diagnosis was clinically suspected, using investigations appropriate to the suspected infection at the discretion of the treating infectious diseases specialist. All patients were managed by the infectious diseases service.

### 2.3 Statistical analysis

Descriptive statistics were used to summarize baseline characteristics. Continuous variables are reported as medians and interquartile ranges (IQRs), whereas categorical variables are presented as counts and percentages with corresponding 95% confidence intervals (95% CIs), calculated using the Wilson score method. Missing data were not imputed. Analyses were performed using available data; denominators for categorical variables and sample sizes for continuous variables therefore varied according to the number of observations available for each variable and time point. Comparisons between groups were performed using appropriate parametric or non-parametric tests according to data distribution. For each patient and laboratory parameter, the post-treatment value was defined as the arithmetic mean of all available measurements obtained after the first intravenous artesunate dose; when only one post-treatment measurement was available, that measurement was used. Paired comparisons of laboratory parameters before and after treatment were performed using the paired *t*-test or Wilcoxon signed-rank test, according to data distribution. All statistical tests were two-sided, and a *p*-value <0.05 was considered statistically significant. Statistical analyses were performed using GraphPad Prism version 10.6.1 (GraphPad Software, San Diego, CA, USA).

### 2.4 Ethics statement

Approval was obtained from the Institutional Ethics Committee for Research of the Hospital Regional de Loreto “Felipe Arriola Iglesias” (CIEI; ID-030-CIEI-2025, May 12, 2025).

## 3 Results

During the study period, 519 laboratory-confirmed malaria diagnoses were recorded at Hospital Regional de Loreto. These comprised 428 cases of *P. vivax* malaria (82.5%), 88 cases of *P. falciparum* malaria (17.0%), two mixed *P. vivax/P. falciparum* infections (0.4%), and one *P. malariae* infection (0.2%). Of the 519 patients, 127 (24.5%) were hospitalized, met the study eligibility criteria, and were included in the analytic cohort. This included 97 of 428 patients with *P. vivax* malaria (22.7%), 27 of 88 with *P. falciparum* malaria (30.7%), two with mixed infection, and one with *P. malariae* infection.

### 3.1 Baseline characteristics of the study population

**Fig. 1:**
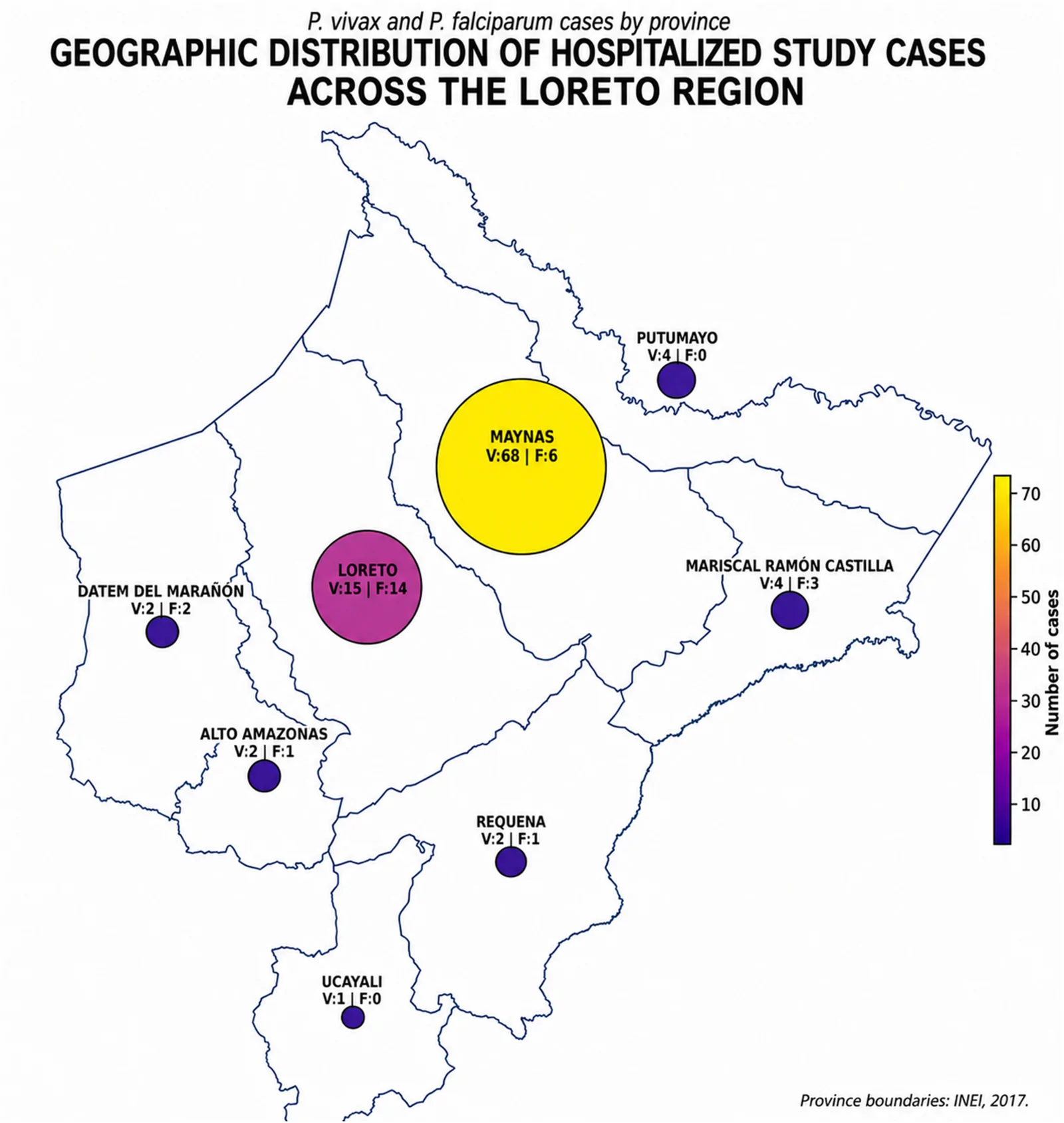
Distribution of study cases across the provinces of the Loreto Region; V: cases of *P. vivax* infection; F: cases of P. falciparum infection.

Our sample included 127 patients, 68 (54%) female, with a median age of 31 years (range 13–87) (Table 1). Most patients (73/127, 57%) were urban or peri-urban residents whilst the remainder were rural. The majority came from Maynas Province, where the Hospital Regional de Loreto is located (75/127, 60%). However, at least one case was recorded from each of the other listed provinces *(*Fig. 1). The median time between symptom onset and hospitalization was 6 days (range 0-15 days). Overall, 67 patients (53%) reported previous malaria episodes. Diabetes mellitus was the most common comorbidity (11/127, 8.7%), while pregnancy was the most frequent recognized predisposing factor (20/127, 15.7%) (Table 1). At least one recognized risk factor or comorbidity was recorded in 42/127 patients (33.1%). Their frequency did not differ between patients with severe and non-severe malaria (22/67 [32.8%] versus 20/60 [33.3%]; Fisher's exact p = 1.00). However, recognized risk factors or comorbidities were significantly more frequent among patients with *P. falciparum* than among those with *P. vivax* malaria (16/27 [59.3%] versus 26/97 [26.8%]; Fisher's exact *p* = 0.0026). This difference largely reflected the greater frequency of pregnancy in the *P. falciparum* group; numbers for individual non-pregnancy comorbidities were too small for meaningful species-specific comparisons (Supplementary Table 1).

**Table 1:** Demographic, clinical, parasitological and laboratory characteristics of the study population (n = 127).

Variable	Overall (n = 127)	Variable	Overall (n = 127)	Variable	Overall (n = 127)
Demographic characteristics, n (%)/median (range)		Predisposing factors		Criteria for severe malaria	
Age	31 (13–87) years	Previous malaria, n (%)	67 (52.8%; 95% CI 44.1–61.2)	Clinical severity criteria^a^, n (%)	50 (39.4%; 95% CI 31.3–48.1)
Female, n (%)	68 (53.5%; 95% CI 44.9–62.0)	**Presence of risk factors or comorbidities, n (%)**		Biochemical severity criteria^b^, n (%)	31 (24.4%; 95% CI 17.8–32.6)
Male, n (%)	59 (46.5%; 95% CI 38.0–55.1)	None	85 (66.9%; 95% CI 58.4–74.5)	**Parasitological characteristics**	
Pregnant women, n (%)	20 (15.7%; 95% CI 10.4–23.1)	One	35 (27.6%; 95% CI 20.5–35.9)	**Plasmodium species, n (%)**	
**Origin, n (%)**		More than one	7 (5.5%; 95% CI 2.7–10.9)	P. falciparum	27 (21.3%; 95% CI 15.0–29.2)
Urban	50 (39.4%; 95% CI 31.3–48.1)	**Presence of risk factors, n (%)**		*P. vivax*	97 (76.4%; 95% CI 68.3–82.9)
Rural	54 (42.5%; 95% CI 34.3–51.2)	Pregnancy	20 (15.7%; 95% CI 10.4–23.1)	Mixed-species malaria or P. malariae	3 (2.4%; 95% CI 0.8–6.7)
Peri-urban	23 (18.1%; 95% CI 12.4–25.7)	HIV	5 (3.9%; 95% CI 1.7–8.9)	**Semi-quantitative parasitaemia (upon entry), n (%)**	
Province, n (%)		Chronic renal failure	1 (0.8%; 95% CI 0.1–4.3)	0 crosses	0 (0.0%; 95% CI 0.0–2.9)
Maynas	75 (59.1%; 95% CI 50.4–67.2)	Cirrhosis/chronic liver disease	1 (0.8%; 95% CI 0.1–4.3)	1 cross	25 (19.7%; 95% CI 13.7–27.4)
Loreto rural	31 (24.4%; 95% CI 17.8–32.6)	Cancer/immunosuppression	0 (0.0%; 95% CI 0.0–2.9)	2 crosses	62 (48.8%; 95% CI 40.3–57.4)
Mariscal Ramón Castilla	6 (4.7%; 95% CI 2.2–9.9)	**Comorbidities**		3 crosses	40 (31.5%; 95% CI 24.1–40.0)
Ucayali	1 (0.8%; 95% CI 0.1–4.3)	Asthma	1 (0.8%; 95% CI 0.1–4.3)	**Time period, median (range)**	
– Alto Amazonas	3 (2.4%; 95% CI 0.8–6.7)	Arterial hypertension	8 (6.3%; 95% CI 3.2–11.9)	Symptom duration before hospitalization	6 (0–15) days
– Putumayo	4 (3.1%; 95% CI 1.2–7.8)	Diabetes	11 (8.7%; 95% CI 4.9–14.8)	Hospital stay	5 (2–18) days
– Requena	3 (2.4%; 95% CI 0.8–6.7)	HCV without cirrhosis	1 (0.8%; 95% CI 0.1–4.3)	Total doses of intravenous artesunate	3 (1–9)
– Datem del Marañón	3 (2.4%; 95% CI 0.8–6.7)	Tuberculosis	1 (0.8%; 95% CI 0.1–4.3)	**Malaria treatment before admission, n (%)**	
– Pebas	1 (0.8%; 95% CI 0.1–4.3)			No treatment	77 (60.6%; 95% CI 51.9–68.7)
				Any treatment	50 (39.4%; 95% CI 31.3–48.1)
				– ACT	3 (2.4%; 95% CI 0.8–6.7)
				– Non-ACT antimalarial treatment	29 (22.8%; 95% CI 16.4–30.9)
				– Empirical treatment without antimalarial activity	18 (14.2%; 95% CI 9.2–21.3)

a Clinical severity criteria included haemorrhage, oedema, renal failure, coma, prostration and shock.

b Biochemical severity criteria included jaundice, severe anaemia, hypoglycaemia, acidosis and hyperlactataemia.

Month of admission showed a seasonal trend, although not statistically significant, even when stratified by *Plasmodium* species or malaria severity. However, *P. vivax* admissions clustered mainly between April and June, peaking in June (12/97, 12%), with an additional increase in December (11/97, 11%). *P. falciparum* cases were fewer and more irregularly distributed (Supplementary Table 1).

At admission, most patients presented with moderate parasite density (two crosses 62/127, 49%), followed by three crosses (40/127, 31%) and one cross (25/127, 20%). *Plasmodium vivax* was identified in 97 patients (76%), followed by *P. falciparum* in 27 (21%); *P. vivax* and *P. falciparum* mixed infection in two and *P. malariae* in one. Overall, 67/127 patients (52.8%) fulfilled at least one WHO severity criterion during hospitalization, while the remainder were hospitalized mainly because of intolerance to oral antimalarial treatment and/or background comorbidities.

### 3.2 Distribution of *Plasmodium* species and disease severity

Species comparisons were restricted to patients with *P. vivax* or *P. falciparum mono-infections*. The proportion of severe malaria was similar between the two species, occurring in 51/97 patients with *P. vivax* (52.6%) and 14/27 patients with *P. falciparum* (51.9%) (Supplementary Table 2). Parasite density at admission was similar between species and across severity groups, with two crosses being the most common baseline grading, and no statistically significant differences by species or severity (Supplementary Table 1).

### 3.3 Pregnancy and malaria characteristics

Twenty pregnant women were included. Malaria infection in this subgroup was predominantly caused by *P. falciparum* (16/20), while *P. vivax* accounted for four cases (p < 0.0001).

### 3.4 WHO severity criteria

**Fig. 2:**
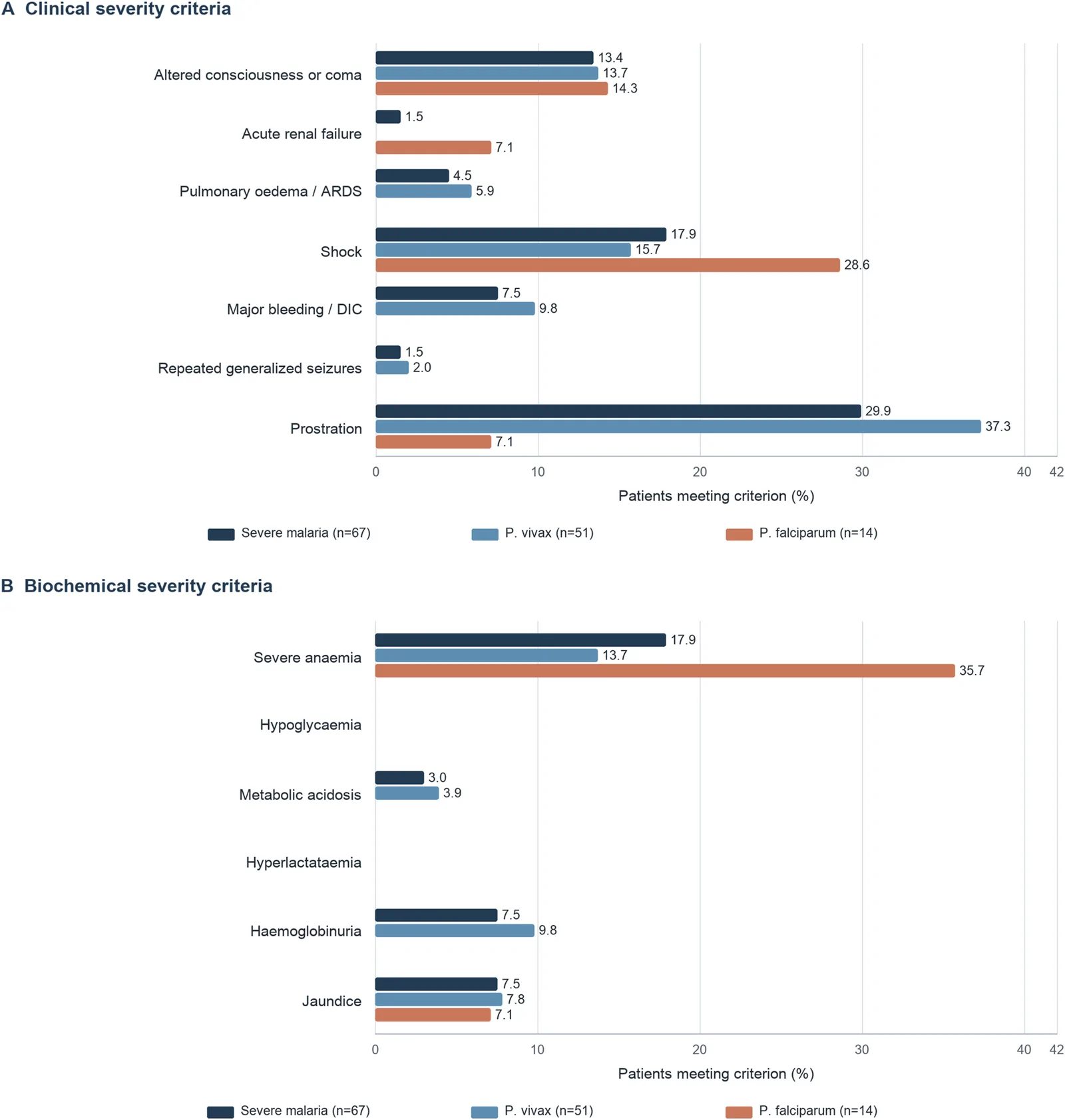
a: Bar charts showing proportions (%) of patients with different clinical criteria for severity*.*Fig. 2b: Bar charts showing proportions (%) of patients with different biochemical criteria for severity. *Definitions are provided* in Appendix Table 1.

Of the 67 patients who fulfilled at least one WHO severity criterion during hospitalization, 66 already met a severity criterion at admission, while one patient first met a criterion during follow-up. Clinical and biochemical severity patterns were broadly comparable between *P. vivax* and *P. falciparum*. Prostration and altered consciousness were the most frequent clinical manifestations, whereas severe anaemia was the most common biochemical criterion (Fig. 2; Supplementary Table 3).

Longitudinal changes in WHO severity manifestations were observed in nine patients: five with *P. vivax*, three with *P. falciparum*, and one with mixed infection. One patient with *P. vivax* fulfilled no severity criterion at admission or during the first 24 h but developed jaundice at ≥24 h, becoming the only patient first classified as severe during follow-up. The other eight patients were already severe at admission. New or recurrent manifestations comprised impaired consciousness, shock, prostration and renal impairment within the first 12 h; metabolic acidosis, jaundice and shock at approximately 12–24 h; and severe anaemia, haemoglobinuria, jaundice and persistent metabolic acidosis at ≥24 h.

### 3.5 Laboratory findings by severity

For baseline laboratory values in the overall population, see Supplementary Table 1*.* At baseline, patients with severe malaria had significantly lower haemoglobin and haematocrit values compared to those with non-severe malaria (median Hb 10.62 versus 11.70 g/dL, p = 0.0073; haematocrit 31.75% versus 34.0%, p = 0.0456). No statistically significant differences between patients affected by severe and non-severe malaria were observed for leukocyte count, platelet count, creatinine, or glucose levels at baseline. After at least one dose of intravenous artesunate, haemoglobin levels measured before discharge were lower in patients with severe malaria compared with non-severe cases (9.50 versus 10.59 g/dL, p = 0.0433), while no significant differences were observed for other parameters.

### 3.6 Laboratory findings according to Plasmodium species

In patients with severe malaria (Supplementary Table 1), *P. falciparum* infection was associated with significantly higher platelet counts (median 131.0 × 10^3^/μL versus 50.0 × 10^3^/μL, p = 0.0020) and lower glucose levels (median 91.42 mg/dL versus 113.1 mg/dL, p = 0.0279) compared with *P. vivax*. No other significant differences were observed.

In non-severe malaria, *P. falciparum* showed a less favourable baseline laboratory profile, with significantly lower Hb and haematocrit levels compared with *P. vivax* (median Hb 10.10 versus 12.30 g/dL, p = 0.0128; haematocrit 30.05% versus 36.65%, p = 0.0042). Platelet counts were significantly higher in *P. falciparum* infections (117.2 × 10^3^/μL versus 68.0 × 10^3^/μL, p = 0.0281), while glucose levels were lower (86.11 mg/dL versus 108.7 mg/dL, p = 0.0060). Among patients classified as non-severe, creatinine values remained within the normal range and were significantly lower in *P. falciparum* than in *P. vivax* malaria (0.47 versus 0.76 mg/dL, p = 0.0001).

### 3.7 Changes in laboratory parameters after treatment

Platelet counts increased substantially after treatment, rising from a median of 69.10 × 10^3^/μL (IQR 36.95–117.50) to 114.3 × 10^3^/μL (IQR 62.50–206.70); (p < 0.0001). Hb and haematocrit values showed modest reductions (Hb: 10.97 to 9.99 g/dL; haematocrit: 32.70% to 30.0% (p = 0.0045 and p = 0.0089, respectively), and leukocyte counts showed small but statistically significant changes, decreasing from 6.5 (IQR 4.50–8.50) to 5.82 (IQR 4.28–7.42) ×10^3^/μL (p = 0.0472).

When stratified by *Plasmodium* species (Supplementary Table 1), no statistically significant differences were observed in post-treatment laboratory parameters between *P. vivax* and *P. falciparum* in patients with severe malaria.

### 3.8 Parasite clearance and clinical outcomes

The median time to parasite clearance following initiation of intravenous artesunate was 48 h (IQR 48–72), with no substantial differences observed according to disease severity.

At 72 h, 89 patients were evaluated, of whom 83 (93.3%) had achieved complete smear clearance, while 6 patients (6.7%) still had detectable parasitaemia. Infecting species included *P. falciparum (3 patients), P. vivax* (2 patients), and mixed *P. falciparum/P. vivax* (1 patient). Three of the six patients were pregnant, IV artesunate was continued in all cases beyond the initial three-dose schedule. Four patients received a total of four IV doses before switching to oral antimalarial therapy, whereas two received eight and nine IV artesunate doses, respectively. All achieved complete parasite clearance at day seven after discharge.

The median hospital stay was 5 days. Two severe malaria patients were hospitalized for 18 days: one patient had a *P. falciparum* infection with severe anaemia and acute renal failure, while the other had a *P. vivax* infection with severe anaemia and prostration. They both received parasitological clearance soon after treatment initiation (with negative microscopy at 48 h).

The only in-hospital death occurred in a pregnant *P. falciparum* malaria patient who was admitted eight days after symptom onset and did not meet clinical or biochemical criteria for severe malaria at admission. Her baseline platelet count was within normal range at 253 × 10^3^/μL, compared with a cohort median of 77.4 × 10^3^/μL (IQR 39.7–124.0). Approximately 48 h after initiation of intravenous artesunate and following three doses, her platelet count had decreased to 36 × 10^3^/μL. At the corresponding assessment, the overall cohort median was 135.5 × 10^3^/μL (IQR 81.3–199.0), while the median was 159 × 10^3^/μL among surviving patients with *P. falciparum* malaria and 187.5 × 10^3^/μL among all surviving pregnant patients. Moderate anaemia persisted throughout hospitalization, with haemoglobin values of 7.3 g/dL at baseline and 7.8 g/dL after three artesunate doses, within a clinical picture of persistent low-level parasitaemia (<1 cross) on day 2. She experienced foetal loss on hospital day 5, followed by severe obstetric haemorrhage, and died the following day despite timely obstetric intervention and transfusion support. The recorded cause of death was massive uterine haemorrhage.

### 3.9 Treatment patterns

Before hospital admission, 67% of patients with severe malaria and 80% of those with non-severe malaria had not received antimalarial treatment. Before hospitalization, 77/127 patients (60.6%) had received no treatment, while 50/127 (39.4%) had received some form of treatment, including ACT in 3/127 (2.4%), non-ACT antimalarial therapy in 29/127 (22.8%), and empirical treatment without antimalarial activity in 18/127 (14.2%) (Table 1).

During hospitalization, all patients received at least one dose of intravenous artesunate at the dosage of 2.4 mg/kg. A complete intravenous artesunate course (at least 3 doses) was administered in 94.1% of severe cases and 90.0% of non-severe cases. Across the sample, the number of intravenous artesunate doses administered ranged from 1 to 9, with a median of 3. Following parenteral treatment, all surviving patients were switched to oral antimalarial therapy.

### 3.10 Coinfections and intestinal parasitosis

Coinfection with dengue was identified in 14 patients, of whom 8 had severe and 6 had non-severe malaria. Other coinfections included two cases of bacterial sepsis and one case of leptospirosis. Intestinal parasitosis was identified by microscopy in 21 patients, of whom 14 with severe and 7 with non-severe malaria.

## 4 Discussion

In this hospital-based study group, *P. vivax* accounted for a substantial burden of severe malaria, while the proportion of severe cases and overall clinical course were broadly comparable between *P. vivax* and *P. falciparum*. Notably, the proportion of severe malaria was comparable between species despite the lower prevalence of recorded risk factors or comorbidities among patients with *P. vivax*, suggesting that the severity observed in this group was not explained simply by a greater comorbidity burden. These findings support the growing recognition that the distinction between malaria species in terms of severity may be less clear-cut than traditionally assumed [11].

Accumulating evidence indicates that *P. vivax*, like *P. falciparum*, can cause substantial haematological alterations, particularly thrombocytopenia and anaemia disproportionate to peripheral parasite density. In some settings, thrombocytopenia is comparable to or more marked than that observed in *P. falciparum* infection [11,22,23]. One possible explanation for the apparent dissociation between circulating parasitaemia and clinical severity is the splenic accumulation of *P. vivax*-infected erythrocytes. Human splenectomy studies have shown that *P. vivax*-infected erythrocytes accumulate with immature reticulocytes in splenic red-pulp compartments. This hidden splenic reservoir may cause peripheral microscopy to underestimate total parasite biomass, thereby potentially contributing to haematological abnormalities despite low circulating parasite densities [24]. Indeed, a greater total parasite biomass has been associated with thrombocytopenia, inflammatory responses, endothelial activation and glycocalyx disruption, implicating microvascular dysfunction in severe *P. vivax* malaria [25].

Endothelial activation is pronounced in acute *P. vivax* malaria and is associated with impaired microvascular reactivity, reduced tissue perfusion and greater disease severity. Reduced L-arginine and nitric oxide bioavailability, glycocalyx degradation and prothrombotic changes may further impair microvascular flow, providing a plausible pathway to organ dysfunction. Unlike in severe *P. falciparum* malaria, endothelial dysfunction in severe *P. vivax* malaria was not associated with increased intravascular haemolysis, suggesting that reduced arginine bioavailability may be more important than haemolysis-mediated nitric oxide depletion [26].

The distribution of severe malaria manifestations in our cohort partly differed from previous reports. Severe anaemia was frequent among patients with *P. vivax* malaria, consistent with systematic reviews [4,11] and with a Brazilian Amazonian series in which severe anaemia and jaundice predominated, while acute respiratory distress syndrome, acute renal failure and shock were also documented [27]. Jaundice, however, was uncommon in our cohort despite being frequently reported in South and Southeast Asian *P. vivax* cohorts [12,28]. The pulmonary oedema/acute respiratory distress syndrome observed in our *P. vivax* subgroup is also consistent with a systematic review that estimated acute respiratory distress syndrome in 2.2% of adults with vivax malaria, with approximately 50% mortality among affected patients [29]. This emphasizes the clinical importance of respiratory involvement despite its relative rarity**.** Neurological manifestations were less prominent than in a paediatric cohort from northern India, where abnormal sensorium and multiple seizures occurred in 50.0% and 44.4% of severe *P. vivax* cases, respectively [30]. Renal and other organ complications described in African severe *P. falciparum* cohorts were also relatively uncommon in our hospitalized series [31,32]. Collectively, these findings suggest that the distribution of WHO severity criteria varies geographically and may change over time within the same setting [23]. Because individual severity criteria carry different prognostic implications [33], multicentre studies are needed to clarify the contributions of transmission intensity, host characteristics and parasite factors.

Finally, the propensity of *P. vivax* to relapse may contribute to severe disease through cumulative rather than exclusively acute mechanisms. Recurrent infections, arising from hepatic hypnozoite activation or from the release of asexual parasites from extravascular reservoirs, may occur before full haematological recovery, leading to progressive anaemia and malnutrition and increasing vulnerability to severe disease during subsequent episodes [34]. Such cumulative morbidity was also proposed to explain findings from Indonesia, where early mortality was higher with *P. falciparum* after the first malaria episode but showed a non-significant trend towards being higher with *P. vivax* after the third [35]. This mechanism may be particularly relevant in the Peruvian Amazon, where *P. vivax* predominates and more than half of our patients reported previous malaria episodes.

Parasite clearance was rapid and comparable across clinical-severity groups, consistent with the established efficacy of intravenous artesunate [36,37]. Although 24.5% of malaria diagnoses recorded at our institution resulted in hospitalization—a substantially higher proportion than the 1.6–3.2% reported in previous Peruvian and Brazilian Amazonian series [27,38]**—** clinical outcomes were generally favourable despite delays between symptom onset and hospitalization and the frequent absence of pre-hospital antimalarial treatment. This apparent contrast suggests that hospitalization in our setting was influenced by referral patterns, admission thresholds and treatment practices and should not be interpreted as equivalent to WHO-defined severe disease. This contrasts with WHO recommendations for prompt initiation of effective antimalarial treatment following diagnosis to reduce progression to severe disease [3]. The single-centre, hospital-based design may therefore have underrepresented the most severe cases: during the study period, some patients may have been too unwell to travel or may have died before reaching the hospital and would consequently not have been captured in our dataset.

How WHO severity criteria are applied throughout hospitalization also warrants consideration. Although the 2024 WHO guidelines do not require formal reassessment against all criteria at fixed intervals, they emphasize prompt recognition of severe manifestations and recommend frequent monitoring of vital signs, level of consciousness and urine output, with repeated blood-glucose assessment when feasible. In our cohort, nine patients developed a WHO-recognized manifestation after the corresponding criterion had been absent at the preceding assessment. Our longitudinal findings support continued clinical and laboratory reassessment throughout hospitalization to detect evolving or recurrent organ dysfunction.

This concern is particularly relevant in pregnancy, a well-recognized risk state for severe malaria because of immunological and physiological changes that increase susceptibility to infection and complications [2,3]. In our cohort, malaria during pregnancy was more frequently caused by *P. falciparum*, consistent with its established association with clinically apparent disease in pregnancy [2]. Although small, this subgroup included several cases of slower parasite clearance and the only recorded death. Importantly, the patient who died did not meet WHO criteria for severe malaria at admission, suggesting that conventional severity classifications may not fully capture the risk of subsequent deterioration in pregnant patients. Although massive uterine haemorrhage was the recorded immediate cause of maternal death, malaria may have contributed to the preceding foetal death, as *P. falciparum* infection during pregnancy is associated with stillbirth [39]. Whether malaria also contributed to the subsequent haemorrhage could not be determined because contemporaneous platelet counts, coagulation parameters and parasite measurements were unavailable, and alternative causes could not be excluded. This case therefore illustrates the difficulty of distinguishing immediate from underlying or contributing causes of maternal death [40].

Previous studies have suggested that dengue–malaria coinfection may be associated with more severe clinical presentations than either infection alone, particularly through hematologic abnormalities [10]. However, given the limited number of coinfected cases in our patient group, we cannot determine whether coinfection increased the risk of severe complications. The *P. vivax* distribution was broadly consistent with expected malaria seasonality in the Peruvian Amazon, where rainfall and humidity favour Anopheles breeding and transmission. However, the December increase was atypical for a simple seasonal pattern and may reflect early rainy-season transmission [20], delayed referral, or *P. vivax* relapse biology.

Other limitations of this study are related to its retrospective design: follow-up laboratory results were incomplete, limiting time-specific and subgroup comparisons. Generally, additional laboratory investigations could have provided a more complete biochemical and microbiological characterization of the cohort. Because blood cultures were obtained only when bacterial coinfection was clinically suspected, undetected invasive bacterial disease may have confounded the attribution of some severe manifestations to malaria. Systematic cultures in future prospective studies could define the local incidence and clarify whether routine testing is warranted. Patient-reported prior malaria history is also an imperfect proxy for true previous exposure, because recall bias and unrecognized asymptomatic infections may have led to exposure misclassification [13]. Regarding laboratory precision, microscopy remains standard in clinical practice but may have reduced sensitivity for low-level or mixed infections [41]. Future studies incorporating PCR-based diagnosis and serological markers of past malaria exposure could improve data accuracy [42].

## 5 Conclusion

*P. vivax* accounted for a substantial burden of WHO-defined severe malaria in this low-transmission Amazonian setting, with a severe-case profile comparable to that of *P. falciparum*. In both species, differentiation between severe and non-severe cases was limited across several clinical, laboratory and parasitological characteristics. Overall, these findings highlight the need to interpret international severity classifications within the local epidemiological context. Given their comparable potential to cause severe disease, both locally prevalent species may warrant similar clinical vigilance.

## CRediT authorship contribution statement

**Livia Bresciani:** Writing – review & editing, Writing – original draft, Visualization, Project administration, Methodology, Investigation, Data curation, Conceptualization. **Mariasilvia Guardiani:** Validation, Software, Formal analysis, Data curation. **Manuel Rojas-Blasquez:** Project administration, Investigation, Data curation. **Edgar Ramirez:** Data collection, Technical support. **Salvatore Iaquinandi:** Resources. **Cesar Martel:** Visualization, Software. **Miguel Velarde-Mera:** Data curation. **Samar Sifuentes-Vidigal:** Data curation. **Anna Carraro:** Supervision. **Gianluca Russo:** Writing – review & editing, Supervision. **Caterina Pasquazzi:** Supervision. **Juan Carlos Celis:** Supervision. **Martin Casapia Morales:** Writing – review & editing, Supervision. **Emanuele Nicastri:** Supervision. **César Ramal Asayag:** Supervision, Resources, Project administration, Conceptualization. **Miriam Lichtner:** Visualization, Supervision, Funding acquisition. **Serena Vita:** Writing – review & editing, Supervision, Project administration, Formal analysis, Conceptualization.

## Data availability statement

The datasets generated and analysed during the current study are not publicly available because they contain sensitive clinical information derived from patient records. De-identified aggregated data and editable source files for figures may be made available from the corresponding author upon reasonable request and subject to institutional approval.

## Funding

This research did not receive any specific grant from funding agencies in the public, commercial, or not-for-profit sectors.

## Declaration of competing interest

The authors declare that they have no known competing financial interests or personal relationships that could have appeared to influence the work reported in this paper.

## Figures and Tables

**Table 1:** WHO criteria for severe malaria in adults and children, according to the WHO Guidelines for malaria (2022).

Category	Adults ≥15 years	Children
**Impaired consciousness**	Glasgow Coma Scale <11 or unarousable coma	Blantyre coma score <3 or unarousable coma
**Prostration**	Inability to sit, stand, or walk without assistance	Inability to sit, stand, walk, breastfeed, or feed, depending on age
**Multiple convulsions**	Two or more convulsions within 24 h	Two or more convulsions within 24 h
**Acidosis/respiratory distress**	Deep acidotic breathing, respiratory distress, lactate >5 mmol/L, or metabolic acidosis	Deep/laboured breathing, respiratory distress, lactate >5 mmol/L, or metabolic acidosis
**Hypoglycaemia**	Blood glucose <2.2 mmol/L/<40 mg/dL	Blood glucose <2.2 mmol/L/<40 mg/dL
**Severe anaemia**	Haemoglobin <7 g/dL or haematocrit <20%	Haemoglobin ≤5 g/dL or haematocrit ≤15%
**Haemoglobinuria**	Dark urine/haemoglobinuria, not explained by another cause	Same
**Jaundice**	Clinical jaundice with evidence of other organ dysfunction	Same; often interpreted with high parasite density or organ dysfunction
**Acute kidney injury**	Creatinine >265 μmol/L/>3.0 mg/dL or severe oliguria/anuria	Same threshold may be used, with clinical assessment of urine output
**Pulmonary oedema/ARDS**	Clinical or radiological evidence of non-cardiogenic pulmonary oedema or ARDS	Same
**Shock**	Systolic blood pressure <80 mmHg with signs of impaired perfusion	Systolic blood pressure <70 mmHg with signs of impaired perfusion
**Significant bleeding/DIC**	Spontaneous bleeding or laboratory evidence of disseminated intravascular coagulation	Same
**Hyperparasitaemia**	P. falciparum parasitaemia >10% of infected erythrocytes	P. falciparum parasitaemia >10% of infected erythrocytes
**Severe *P. vivax* malaria**	Severe malaria criteria apply, but no parasite-density threshold is defined	Same
**Severe *P. knowlesi* malaria**	Parasite density >100,000/μL may indicate severe disease	Same

Abbreviations: ARDS, acute respiratory distress syndrome; DIC, disseminated intravascular coagulation; WHO, World Health Organization.

## References

[1] World Health Organization (2025).

[2] Poespoprodjo J.R., Douglas N.M., Ansong D., Kho S., Anstey N.M. (2023). Malaria. Lancet.

[3] World Health Organization (2022).

[4] Singh D., Uddin A., Bajaj K., Chaturvedi A., Garg R., Chandra K. (2025). Clinical spectrum and complications of Plasmodium vivax malaria: a retrospective study from Delhi, India. Malar J.

[5] Lacerda M.V.G., Fragoso S.C.P., Alecrim M.G.C., Alexandre M.A.A., Magalhães B.M.L., Siqueira A.M. (2012). Postmortem characterization of patients with clinical diagnosis of Plasmodium vivax malaria: to what extent does this parasite kill?. Clin Infect Dis.

[6] Gehlawat V.K., Arya V., Kaushik J.S., Gathwala G. (2013). Clinical spectrum and treatment outcome of severe malaria caused by Plasmodium vivax in 18 children from northern India. Pathog Glob Health.

[7] Global Technical Strategy for Malaria 2016-2030 (2021).

[8] Centro Nacional de Epidemiología P y C de E– M de S del Perú (2024). Boletín epidemiológico SE 52 – 2024. Lima.

[9] Vasconcelos M.P.A., Sánchez-Arcila J.C., Peres L., de Sousa P.S.F., dos Santos Alvarenga M.A., Castro-Alves J. (2023). Malarial and intestinal parasitic co-infections in indigenous populations of the Brazilian amazon rainforest. J Infect Public Health.

[10] Magalhães B.M.L., Siqueira A.M., Alexandre M.A.A., Souza M.S., Gimaque J.B., Bastos M.S. (2014). P. vivax malaria and dengue fever Co-infection: a cross-sectional study in the Brazilian amazon. PLoS Neglected Trop Dis.

[11] Naing Mawvnwjwm Cho (2014). Is Plasmodium vivax malaria a severe malaria?: a systematic review and meta-analysis. LoS NeglTrop Dis.

[12] Kotepui M., Kotepui K.U., Milanez G.D.J., Masangkay F.R. (2020). Prevalence and risk factors related to poor outcome of patients with severe Plasmodium vivax infection: a systematic review, meta-analysis, and analysis of case reports. BMC Infect Dis.

[13] Fernandez-Miñope Carlos (2021). Towards one standard treatment for uncomplicated Plasmodium falciparum and Plasmodium vivax malaria: perspectives from and for the Peruvian Amazon. Int J Infect Dis.

[14] Carrasco-Escobar G., Gamboa D., Castro M.C., Bangdiwala S.I., Rodriguez H., Contreras-Mancilla J. (2017). Micro-epidemiology and spatial heterogeneity of P. vivax parasitaemia in riverine communities of the Peruvian Amazon: a multilevel analysis. Sci Rep.

[15] Kattenberg J.H., Fernandez-Miñope C., van Dijk N.J., Llacsahuanga Allcca L., Guetens P., Valdivia H.O. (2023). Malaria molecular surveillance in the Peruvian Amazon with a novel highly multiplexed Plasmodium falciparum AmpliSeq assay. Microbiol Spectr.

[16] Garnelo L., Parente R.C.P., Puchiarelli M.L.R., Correia P.C., Torres M.V., Herkrath F.J. (2020). Barriers to access and organization of primary health care services for rural riverside populations in the Amazon. Int J Equity Health.

[17] Halsey E.S., Baldeviano G.C., Edgel K.A., Vilcarromero S., Sihuincha M., Lescano A.G. (2016). Symptoms and Immune markers in Plasmodium/dengue virus co-infection compared with mono-infection with either in Peru. PLoS Neglected Tropical Diseases..

[18] de Albuquerque N.R.M. (2025). The source-sink dynamics of Plasmodium vivax may undermine malaria elimination efforts in the Amazon: an epidemiological and population genomic study. J Infect Dis.

[19] Bôtto-Menezes C., Bardají A., dos Santos Campos G., Fernandes S., Hanson K., Martínez-Espinosa F.E. (2016). Costs associated with malaria in pregnancy in the Brazilian Amazon, a low endemic area where Plasmodium vivax predominates. PLoS Neglected Trop Dis.

[20] Segala F.V., Guido G., Stroffolini G., Masini L., Cattaneo P., Moro L. (2025). Insights into the ecological and climate crisis: emerging infections threatening human health. Acta Trop.

[21] WHO. Basic (2010).

[22] Wynberg E., Commons R.J., Humphreys G., Ashurst H., Burrow R., Adjei G.O. (2023). Variability in white blood cell count during uncomplicated malaria and implications for parasite density estimation: a WorldWide Antimalarial resistance network individual patient data meta-analysis. Malar J.

[23] Acharya J., Harwani D. (2022). Changing pattern of severe manifestations of Plasmodium falciparum and Plasmodium vivax malaria. J Vector Borne Dis.

[24] Kho S., Qotrunnada L., Leonardo L., Andries B., Wardani P.A.I., Fricot A. (2021). Evaluation of splenic accumulation and colocalization of immature reticulocytes and Plasmodium vivax in asymptomatic malaria: a prospective human splenectomy study. PLoS Med.

[25] Silva-Filho J.L., Dos-Santos J.C., Judice C., Beraldi D., Venugopal K., Lima D. (2021). Total parasite biomass but not peripheral parasitaemia is associated with endothelial and haematological perturbations in Plasmodium vivax patients. eLife.

[26] Barber B.E., William T., Grigg M.J., Piera K.A., Chen Y., Wang H. (2016). Nitric oxide–dependent endothelial dysfunction and reduced arginine bioavailability in Plasmodium vivax malaria but no greater increase in intravascular hemolysis in severe disease. J Infect Dis.

[27] Alexandre M.A., Ferreira C.O., Siqueira A.M., Magalhães B.L., Mourão M.P.G., Lacerda M.V. (2010). Severe Plasmodium vivax malaria, Brazilian Amazon. Emerg Infect Dis.

[28] Duong M.C., Pham O.K.N., Thai T.T., Lee R., Nguyen T.P., Nguyen V.V.C. (2023). Magnitude and patterns of severe Plasmodium vivax monoinfection in Vietnam: a 4-year single-center retrospective study. Front Med.

[29] Val F., Machado K., Barbosa L., Salinas J.L., Siqueira A.M., Costa Alecrim M.G. (2017). Respiratory complications of Plasmodium vivax malaria: systematic review and meta-analysis. Am J Trop Med Hyg.

[30] Kojom Foko L.P., Arya A., Sharma A., Singh V. (2021). Epidemiology and clinical outcomes of severe Plasmodium vivax malaria in India. J Infect.

[31] Dondorp (2010). Artesunate versus quinine in the treatment of severe falciparum malaria in African children (AQUAMAT). Lancet.

[32] Conroy A.L., Hawkes M.T., Leligdowicz A., Mufumba I., Starr M.C., Zhong K. (2022). Blackwater fever and acute kidney injury in children hospitalized with an acute febrile illness: pathophysiology and prognostic significance. BMC Med.

[33] Balerdi-Sarasola Leire (2024). Not all severe malaria cases are severe: is it time to redefine severity criteria for malaria in non-endemic regions?. Trav Med Infect Dis.

[34] Anstey N.M., Tham W.-H., Shanks G.D., Poespoprodjo J.R., Russell B.M., Kho S. (2024). The biology and pathogenesis of vivax malaria. Trends Parasitol.

[35] Dini S., Douglas N.M., Poespoprodjo J.R., Kenangalem E., Sugiarto P., Plumb I.D. (2020). The risk of morbidity and mortality following recurrent malaria in Papua, Indonesia: a retrospective cohort study. BMC Med.

[36] Byakika-Kibwika P., Nyakato P., Lamorde M., Kiragga A.N. (2018). Assessment of parasite clearance following treatment of severe malaria with intravenous artesunate in Ugandan children enrolled in a randomized controlled clinical trial. Malar J.

[37] White N. (2011). The parasite clearance curve. Malar J.

[38] Quispe A.M., Pozo E., Guerrero E., Durand S., Baldeviano G.C., Edgel K.A. (2014). Plasmodium vivax hospitalizations in a monoendemic malaria Region: severe Vivax malaria?. The American Society of Tropical Medicine and Hygiene.

[39] Moore K.A., Simpson J.A., Scoullar M.J.L., McGready R., Fowkes F.J.I. (2017). Quantification of the association between malaria in pregnancy and stillbirth: a systematic review and meta-analysis. Lancet Global Health.

[40] Kourtis A.P., Read J.S., Jamieson D.J. (2014). Pregnancy and infection. N Engl J Med.

[41] World Health Organization (1988). Malaria diagnosis: memorandum from a WHO meeting. Bull World Health Organ.

[42] Tayipto Y., Liu Z., Mueller I., Longley R.J. (2022). Serology for Plasmodium vivax surveillance: a novel approach to accelerate towards elimination. Parasitol Int.

