# Supplementary material for: Comparable severity of Plasmodium vivax and Plasmodium falciparum malaria in hospitalized patients in the Peruvian Amazon
Source: New Microbes New Infect. 2026 Sep 8;73:101849. doi: 10.1016/j.nmni.2026.101849 (PMC13589080; doi:10.1016/j.nmni.2026.101849)
Supplement: Supplementary file 1 [file mmc1.docx]

**Supplementary material**

**Supplementary Table 1.** Demographic, clinical, parasitological and laboratory characteristics of the study population (n = 127). Pregnancy was included among the risk factors. Mixed infections (n = 2) were excluded from analyses by infecting species.

| **Variable** | **P. vivax (n=97)** | **P. falciparum (n=27)** | **P value (species)** | **Severe malaria (n=67)** | **Non-severe malaria (n=60)** | **P value (severity)** |
| --- | --- | --- | --- | --- | --- | --- |
| **Demographic characteristics** | | | | | | |
| Female sex, n (%) | 45 (46.4%; 95% CI 36.7–56.3) | 22 (81.5%; 95% CI 63.3–91.8) | ns* | 38 (56.7%; 95% CI 44.7–67.9) | 30 (50.0%; 95% CI 37.7–62.3) | ns |
| Male sex, n (%) | 52 (53.6%; 95% CI 43.7–63.3) | 5 (18.5%; 95% CI 8.2–36.7) | ns | 29 (43.3%; 95% CI 32.1–55.3) | 30 (50.0%; 95% CI 37.7–62.3) | ns |
| Pregnant women, n (%) | 4 (4.1%; 95% CI 1.6–10.1) | 16 (59.3%; 95% CI 40.7–75.5) | ns | 11 (16.4%; 95% CI 9.4–27.1) | 9 (15.0%; 95% CI 8.1–26.1) | ns |
| Overall P value |  |  |  |  |  | ns |
| **Origin, n (%)** | | | | | | |
| Urban | 45 (46.4%; 95% CI 36.7–56.3) | 4 (14.8%; 95% CI 5.9–32.5) | ns | 23 (34.3%; 95% CI 24.0–46.2) | 27 (45.0%; 95% CI 33.1–57.5) | ns |
| Rural | 35 (36.1%; 95% CI 27.2–46.1) | 17 (63.0%; 95% CI 44.9–78.2) | ns | 30 (44.8%; 95% CI 33.7–56.5) | 24 (40.0%; 95% CI 28.4–52.9) | ns |
| Peri-urban | 17 (17.5%; 95% CI 11.2–26.4) | 6 (22.2%; 95% CI 10.6–40.8) | ns | 14 (20.9%; 95% CI 13.2–31.5) | 9 (15.0%; 95% CI 8.1–26.1) | ns |
| Overall P value |  |  |  |  |  | ns |
| **Province, n (%)** | | | | | | |
| Maynas | 68 (70.1%; 95% CI 60.4–78.3) | 6 (22.2%; 95% CI 10.6–40.8) | ns | 37 (55.2%; 95% CI 43.3–66.4) | 38 (63.3%; 95% CI 50.7–74.3) | ns |
| Loreto rural | 15 (15.5%; 95% CI 9.6–24.1) | 14 (51.9%; 95% CI 34.0–69.3) | ns | 17 (25.4%; 95% CI 16.5–36.9) | 14 (23.3%; 95% CI 14.8–34.8) | ns |
| Mariscal Ramón Castilla | 3 (3.1%; 95% CI 1.1–8.8) | 3 (11.1%; 95% CI 3.9–28.1) | ns | 4 (6.0%; 95% CI 2.3–14.4) | 2 (3.3%; 95% CI 0.9–11.3) | ns |
| Ucayali | 1 (1.0%; 95% CI 0.2–5.6) | 0 (0.0%; 95% CI 0.0–12.5) | ns | 1 (1.5%; 95% CI 0.3–8.0) | 0 (0.0%; 95% CI 0.0–6.0) | ns |
| Alto Amazonas | 2 (2.1%; 95% CI 0.6–7.2) | 1 (3.7%; 95% CI 0.7–18.3) | ns | 1 (1.5%; 95% CI 0.3–8.0) | 2 (3.3%; 95% CI 0.9–11.3) | ns |
| Putumayo | 4 (4.1%; 95% CI 1.6–10.1) | 0 (0.0%; 95% CI 0.0–12.5) | ns | 3 (4.5%; 95% CI 1.5–12.5) | 1 (1.7%; 95% CI 0.3–8.9) | ns |
| Requena | 2 (2.1%; 95% CI 0.6–7.2) | 1 (3.7%; 95% CI 0.7–18.3) | ns | 2 (3.0%; 95% CI 0.8–10.4) | 1 (1.7%; 95% CI 0.3–8.9) | ns |
| Datem del Marañón | 1 (1.0%; 95% CI 0.2–5.6) | 2 (7.4%; 95% CI 2.1–23.4) | ns | 2 (3.0%; 95% CI 0.8–10.4) | 1 (1.7%; 95% CI 0.3–8.9) | ns |
| Pebas | 1 (1.0%; 95% CI 0.2–5.6) | 0 (0.0%; 95% CI 0.0–12.5) | ns | 0 (0.0%; 95% CI 0.0–5.4) | 1 (1.7%; 95% CI 0.3–8.9) | ns |
| Overall P value |  |  |  |  |  | ns |
| **Month of admission, n (%)** | | | | | | |
| January | 3 (3.1%; 95% CI 1.1–8.8) | 0 (0.0%; 95% CI 0.0–12.5) | ns | 3 (4.5%; 95% CI 1.5–12.5) | 0 (0.0%; 95% CI 0.0–6.0) | ns |
| February | 5 (5.2%; 95% CI 2.2–11.6) | 1 (3.7%; 95% CI 0.7–18.3) | ns | 2 (3.0%; 95% CI 0.8–10.4) | 4 (6.7%; 95% CI 2.6–16.1) | ns |
| March | 9 (9.3%; 95% CI 5.0–16.8) | 5 (18.5%; 95% CI 8.2–36.7) | ns | 10 (14.9%; 95% CI 8.3–25.3) | 4 (6.7%; 95% CI 2.6–16.1) | ns |
| April | 11 (11.3%; 95% CI 6.5–19.0) | 3 (11.1%; 95% CI 3.9–28.1) | ns | 7 (10.4%; 95% CI 5.2–20.0) | 7 (11.7%; 95% CI 5.8–22.2) | ns |
| May | 11 (11.3%; 95% CI 6.5–19.0) | 4 (14.8%; 95% CI 5.9–32.5) | ns | 9 (13.4%; 95% CI 7.2–23.5) | 7 (11.7%; 95% CI 5.8–22.2) | ns |
| June | 12 (12.4%; 95% CI 7.3–20.3) | 3 (11.1%; 95% CI 3.9–28.1) | ns | 4 (6.0%; 95% CI 2.3–14.4) | 11 (18.3%; 95% CI 10.6–29.8) | ns |
| July | 8 (8.2%; 95% CI 4.2–15.4) | 2 (7.4%; 95% CI 2.1–23.4) | ns | 4 (6.0%; 95% CI 2.3–14.4) | 6 (10.0%; 95% CI 4.7–20.1) | ns |
| August | 10 (10.3%; 95% CI 5.7–18.0) | 3 (11.1%; 95% CI 3.9–28.1) |  | 9 (13.4%; 95% CI 7.2–23.5) | 4 (6.7%; 95% CI 2.6–16.1) |  |
| September | 5 (5.2%; 95% CI 2.2–11.6) | 4 (14.8%; 95% CI 5.9–32.5) |  | 5 (7.5%; 95% CI 3.3–16.3) | 5 (8.3%; 95% CI 3.6–18.1) |  |
| October | 5 (5.2%; 95% CI 2.2–11.6) | 1 (3.7%; 95% CI 0.7–18.3) |  | 3 (4.5%; 95% CI 1.5–12.5) | 3 (5.0%; 95% CI 1.7–13.7) |  |
| November | 7 (7.2%; 95% CI 3.6–14.1) | 1 (3.7%; 95% CI 0.7–18.3) |  | 5 (7.5%; 95% CI 3.3–16.3) | 4 (6.7%; 95% CI 2.6–16.1) |  |
| December | 11 (11.3%; 95% CI 6.5–19.0) | 0 (0.0%; 95% CI 0.0–12.5) |  | 6 (9.0%; 95% CI 4.2–18.3) | 5 (8.3%; 95% CI 3.6–18.1) |  |
| Overall P value |  |  |  |  |  | ns |
| **Patient prior history** | | | | | | |
| Previous malaria, n (%) | 52 (53.6%; 95% CI 43.7–63.3) | 12 (44.4%; 95% CI 27.9–62.2) | ns | 37 (55.2%; 95% CI 43.3–66.4) | 30 (50.0%; 95% CI 37.7–62.3) | ns |
| **Presence of risk factors or comorbidities**, n (%)** | | | | | | |
| None | 71 (73.2%; 95% CI 63.6–81.0) | 11 (40.7%; 95% CI 24.5–59.3) | - | 45 (67.2%; 95% CI 55.2–77.2) | 40 (66.7%; 95% CI 54.1–77.2) | - |
| One | 20 (20.6%; 95% CI 13.8–29.6) | 15 (55.6%; 95% CI 37.8–72.1) | - | 17 (25.4%; 95% CI 16.5–36.9) | 18 (30.0%; 95% CI 19.9–42.7) | - |
| More than one | 6 (6.2%; 95% CI 2.9–12.9) | 1 (3.7%; 95% CI 0.7–18.3) | - | 5 (7.5%; 95% CI 3.3–16.3) | 2 (3.3%; 95% CI 0.9–11.3) | - |
| Overall comparison: none vs ≥1 |  |  | 0.0026 |  |  | 1.00 |
| **Types of risk factors** | | | | | | |
| Pregnancy | 4 (4.1%; 95% CI 1.6–10.1) | 16 (59.3%; 95% CI 40.7–75.5) | <0.0001 | 11 (16.4%; 95% CI 9.4–27.1) | 9 (15.0%; 95% CI 8.1–26.1) | ns |
| HIV | 4 (4.1%; 95% CI 1.6–10.1) | 1 (3.7%; 95% CI 0.7–18.3) | ns | 2 (3.0%; 95% CI 0.8–10.4) | 3 (5.0%; 95% CI 1.7–13.7) | ns |
| Chronic renal failure | 1 (1.0%; 95% CI 0.2–5.6) | 0 (0.0%; 95% CI 0.0–12.5) | ns | 0 (0.0%; 95% CI 0.0–5.4) | 1 (1.7%; 95% CI 0.3–8.9) | ns |
| Cirrhosis / chronic liver disease | 1 (1.0%; 95% CI 0.2–5.6) | 0 (0.0%; 95% CI 0.0–12.5) | ns | 0 (0.0%; 95% CI 0.0–5.4) | 1 (1.7%; 95% CI 0.3–8.9) | ns |
| Cancer / immunosuppression | 0 (0.0%; 95% CI 0.0–3.8) | 0 (0.0%; 95% CI 0.0–12.5) | ns | 0 (0.0%; 95% CI 0.0–5.4) | 0 (0.0%; 95% CI 0.0–6.0) | ns |
| **Types of comorbidities** | | | | | | |
| Asthma | 1 (1.0%; 95% CI 0.2–5.6) | 0 (0.0%; 95% CI 0.0–12.5) | ns | 1 (1.5%; 95% CI 0.3–8.0) | 0 (0.0%; 95% CI 0.0–6.0) | ns |
| Arterial hypertension | 8 (8.2%; 95% CI 4.2–15.4) | 0 (0.0%; 95% CI 0.0–12.5) | ns | 5 (7.5%; 95% CI 3.3–16.3) | 3 (5.0%; 95% CI 1.7–13.7) | ns |
| Diabetes | 11 (11.3%; 95% CI 6.5–19.0) | 0 (0.0%; 95% CI 0.0–12.5) | ns | 6 (9.0%; 95% CI 4.2–18.3) | 5 (8.3%; 95% CI 3.6–18.1) | ns |
| HCV without cirrhosis | 1 (1.0%; 95% CI 0.2–5.6) | 0 (0.0%; 95% CI 0.0–12.5) | ns | 1 (1.5%; 95% CI 0.3–8.0) | 0 (0.0%; 95% CI 0.0–6.0) | ns |
| Tuberculosis | 1 (1.0%; 95% CI 0.2–5.6) | 0 (0.0%; 95% CI 0.0–12.5) | ns | 1 (1.5%; 95% CI 0.3–8.0) | 0 (0.0%; 95% CI 0.0–6.0) | ns |
| **Criteria for severe malaria** | | | | | | |
| Clinical severity criteria***, n (%) | 41 (42.3%; 95% CI 33.0–52.1) | 8 (29.6%; 95% CI 15.9–48.5) | ns | 50 (74.6%; 95% CI 63.0–83.5) | 0 (0.0%; 95% CI 0.0–6.0) | ns |
| Biochemical severity criteria****, n (%) | 22 (22.7%; 95% CI 15.6–31.9) | 8 (29.6%; 95% CI 15.9–48.5) | ns | 31 (46.3%; 95% CI 35.1–57.8) | 0 (0.0%; 95% CI 0.0–6.0) | ns |
| **Semi-quantitative parasitaemia prior to treatment with intravenous Artesunate** | | | | | | |
| 0 crosses | 0 (0.0%; 95% CI 0.0–3.8) | 0 (0.0%; 95% CI 0.0–12.5) | ns | 0 (0.0%; 95% CI 0.0–5.4) | 0 (0.0%; 95% CI 0.0–6.0) | ns |
| 1 cross | 16 (16.5%; 95% CI 10.4–25.1) | (16.5%; 95% CI 10.4–25.1)8 (29.6%; 95% CI 15.9–48.5) | ns | 12 (17.9%; 95% CI 10.6–28.7) | (17.9%; 95% CI 10.6–28.7)13 (21.7%; 95% CI 13.5–33.1) | ns |
| 2 crosses | 50 (51.5%; 95% CI 41.7–61.2) | 11 (40.7%; 95% CI 24.5–59.3) | ns | 36 (53.7%; 95% CI 42.0–65.0) | 26 (43.3%; 95% CI 31.7–55.8) | ns |
| 3 crosses | 31 (32.0%; 95% CI 23.5–41.9) | 8 (29.6%; 95% CI 15.9–48.5) | ns | 19 (28.4%; 95% CI 18.8–40.4) | 21 (35.0%; 95% CI 24.2–47.7) | ns |
| **Semi-quantitative parasitaemia following treatment with intravenous Artesunate (at least one dose)** | | | | | | |
| 0 crosses | 63 (64.9%; 95% CI 55.0–73.7) | 13 (48.1%; 95% CI 30.7–66.0) | ns | 43 (64.2%; 95% CI 52.2–74.6) | 36 (60.0%; 95% CI 47.4–71.4) | ns |
| 1 cross | 0 (0.0%; 95% CI 0.0–3.8) | 5 (18.5%; 95% CI 8.2–36.7) | ns | 4 (6.0%; 95% CI 2.3–14.4) | 1 (1.7%; 95% CI 0.3–8.9) | ns |
| 2 crosses | 0 (0.0%; 95% CI 0.0–3.8) | 0 (0.0%; 95% CI 0.0–12.5) | ns | 1 (1.5%; 95% CI 0.3–8.0) | 2 (3.3%; 95% CI 0.9–11.3) | ns |
| 3 crosses | 3 (3.1%; 95% CI 1.1–8.8) | 0 (0.0%; 95% CI 0.0–12.5) | ns | 0 (0.0%; 95% CI 0.0–5.4) | 0 (0.0%; 95% CI 0.0–6.0) | ns |
| **Laboratory parameters prior to treatment with intravenous Artesunate** | | | | | | |
| Haemoglobin (g/dL), median (IQR) | 11.37 (9.61–13.40) | 10.30 (7.30–11.20) | 0.0081 | 10.62 (7.20–12.10) | 11.70 (9.80–13.43) | 0.0073 |
| Haematocrit (%), median (IQR) | 33.80 (28.60–39.80) | 31.50 (22.50–34.0) | 0.0159 | 31.75 (23.90–36.03) | 34.0 (28.40–39.80) | 0.0456 |
| Leukocytes (×10³/µL), median (IQR) | 6.63 (4.51–8.54) | 6.06 (4.35–7.71) | ns | 6.53 (5.30–8.36) | 6.62 (3.99–8.71) | ns |
| Platelets (×10³/µL), median (IQR) | 57.65 (33.50–95.0) | 131.0 (64.0–211.0) | 0.0001 | 68.0 (36.80–122.0) | 73.30 (37.0–113.0) | ns |
| Creatinine (mg/dL), median (IQR) | 0.80 (0.60–0.99) | 0.53 (0.46–0.65) | <0.0001 | 0.80 (0.60–0.98) | 0.70 (0.50–0.95) | ns |
| Glucose (mg/dL), median (IQR) | 111.1 (94.09–130.0) | 90.63 (80.32–107.4) | 0.0003 | 107.9 (89.80–125.70) | 105.0 (89.98–122.0) | ns |
| **Laboratory parameters following treatment with intravenous Artesunate (at least one dose)** | | | | | | |
| Haemoglobin (g/dL), median (IQR) | 10.11 (8.06–11.70) | 9.45 (8.10–10.23) | ns | 9.50 (7.90–10.96) | 10.59 (8.15–11.94) | 0.0433 |
| Haematocrit (%), median (IQR) | 30.0 (25.0–35.50) | 29.50 (26.40–31.60) | ns | 29.20 (25.40–33.40) | 32.10 (25.05–36.30) | ns |
| Leukocytes (×10³/µL), median (IQR) | 5.69 (4.31–7.26) | 6.49 (3.77–7.63) | ns | 6.03 (4.22–7.59) | 5.67 (4.43–7.28) | ns |
| Platelets (×10³/µL), median (IQR) | 102.2 (57.50–176.80) | 154.5 (107.0–258.5) | 0.0157 | 99.0 (56.05–206.1) | 124.0 (73.20–214.2) | ns |
| Creatinine (mg/dL), median (IQR) | 0.70 (0.58–0.85) | 0.48 (0.45–0.59) | 0.0002 | 0.64 (0.55–0.85) | 0.68 (0.50–0.82) | ns |
| Glucose (mg/dL), median (IQR) | 100.0 (90.58–117.40) | 88.67 (69.22–98.0) | 0.0315 | 107.9 (89.80–125.70) | 96.75 (85.19–110.1) | ns |
| **Treatment characteristics during hospital stay: Intravenous medication** | | | | | | |
| Artesunate + clindamycin, n (%) | 69 (71.1%; 95% CI 61.4–79.2) | 21 (77.8%; 95% CI 59.2–89.4) | ns | 50 (74.6%; 95% CI 63.1–83.5) | 42 (70.0%; 95% CI 57.5–80.1) | ns |
| Artesunate alone, n (%) | 28 (28.9%; 95% CI 20.8–38.6) | 6 (22.2%; 95% CI 10.6–40.8) | ns | 17 (25.4%; 95% CI 16.5–36.9) | 18 (30.0%; 95% CI 19.9–42.5) | ns |
| Total artesunate doses =/>3, n (%) | 89 (91.8%; 95% CI 84.5–95.8) | 25 (92.6%; 95% CI 76.6–97.9) | ns | 63 (94.0%; 95% CI 85.8–97.7) | 54 (90.0%; 95% CI 79.9–95.3) | ns |
| **Treatment characteristics during hospital stay: Oral medication******* | | | | | | |
| ACT****** administered, n (%) | 12 (12.4%; 95% CI 7.2–20.4) | 3 (11.1%; 95% CI 3.9–28.1) | ns | 8 (11.9%; 95% CI 6.2–21.5) | 8 (13.3%; 95% CI 6.9–24.1) | ns |
| Primaquine administered, n (%) | 57 (58.8%; 95% CI 48.9–68.1) | 7 (25.9%; 95% CI 13.2–44.7) | ns | 32 (47.8%; 95% CI 36.4–59.5) | 32 (53.3%; 95% CI 40.9–65.3) | ns |

** ns = non-significant.*

*** Comorbidities were conditions that did not meet the WHO criteria for risk factors for severe malaria.*

**** Clinical severity criteria were defined according to the WHO Guidelines for malaria (2022).*

***** Biochemical severity criteria were defined according to the WHO Guidelines for malaria (2022).*

****** Oral medication was administered during the hospital stay after the final dose of intravenous artesunate. This does not refer to medication administered after hospital discharge, although the choice of medication may have overlapped.*

******* ACT = artemisinin-based combination therapy.*

**Supplementary Table 2.** Association between Plasmodium species and severe malaria or pregnancy. Values are presented as n (%) with 95% confidence intervals. Pregnancy analysis was restricted to female participants. Mixed infections (n=2) and the P. malariae infection (n=1) were excluded from analyses by infecting species.

| **Outcome** | **P. vivax (n=97)** | **P. falciparum (n=27)** | **PR (95% CI)** | **p value** |
| --- | --- | --- | --- | --- |
| Severe malaria | 51 (52.6%; 95% CI 42.7–62.3) | 14 (51.9%; 95% CI 34.0–69.3) | 0.99 (0.66–1.49) | 0.83 |
| Non-severe malaria | 46 (47.4%; 95% CI 37.7–57.3) | 13 (48.1%; 95% CI 30.7–66.0) | - | - |
| Pregnant* | 4 (8.9%; 95% CI 3.5–20.7) | 16 (72.7%; 95% CI 52.4–86.5) | 8.18 (3.18–21.04) | <0.0001 |
| Non-pregnant* | 41 (91.1%; 95% CI 79.3–96.5) | 6 (27.3%; 95% CI 13.5–47.6) | - | - |

** Pregnancy analysis was restricted to female participants (45 women with P. vivax and 22 women with P. falciparum).*

*Abbreviations: CI, confidence interval; PR, prevalence ratio.*

**Supplementary Table 3.** Clinical and biochemical severity criteria among patients with severe malaria according to infecting Plasmodium species.

| **Severity criteria** | **Severe malaria (n=67)** | **P. vivax (n=51)** | **P. falciparum (n=14)** |
| --- | --- | --- | --- |
| **Clinical severity criteria** | | | |
| None | 17 (25.4%; 95% CI 16.5–36.9) | 10 (19.6%; 95% CI 11.0–32.5) | 6 (42.9%; 95% CI 21.4–67.4) |
| Altered level of consciousness or coma (GCS <11 or Blantyre <3) | 9 (13.4%; 95% CI 7.2–23.6) | 7 (13.7%; 95% CI 6.8–25.7) | 2 (14.3%; 95% CI 4.0–39.9) |
| Acute renal failure (creatinine >3 mg/dL or oliguria/anuria) | 1 (1.5%; 95% CI 0.3–8.0) | 0 (0.0%; 95% CI 0.0–7.0) | 1 (7.1%; 95% CI 1.3–31.5) |
| Pulmonary oedema / acute respiratory distress syndrome (ARDS) | 3 (4.5%; 95% CI 1.5–12.4) | 3 (5.9%; 95% CI 2.0–15.9) | 0 (0.0%; 95% CI 0.0–21.5) |
| Shock (SBP <70 mmHg in children or <80 mmHg in adults, with evidence of impaired perfusion) | 12 (17.9%; 95% CI 10.6–28.7) | 8 (15.7%; 95% CI 8.2–28.0) | 4 (28.6%; 95% CI 11.7–54.6) |
| Major bleeding / disseminated intravascular coagulation (DIC) | 5 (7.5%; 95% CI 3.2–16.3) | 5 (9.8%; 95% CI 4.3–21.0) | 0 (0.0%; 95% CI 0.0–21.5) |
| Repeated generalized seizures (≥2 seizures/24 h) | 1 (1.5%; 95% CI 0.3–8.0) | 1 (2.0%; 95% CI 0.3–10.3) | 0 (0.0%; 95% CI 0.0–21.5) |
| Prostration (inability to sit, stand, or walk without assistance) | 20 (29.9%; 95% CI 20.2–41.7) | 19 (37.3%; 95% CI 25.3–51.0) | 1 (7.1%; 95% CI 1.3–31.5) |
| **Biochemical severity criteria** | | | |
| None | 36 (53.7%; 95% CI 41.9–65.1) | 29 (56.9%; 95% CI 43.3–69.5) | 6 (42.9%; 95% CI 21.4–67.4) |
| Severe anaemia | 14 (20.9%; 95% CI 12.9–32.1) | 9 (17.6%; 95% CI 9.7–30.2) | 4 (28.6%; 95% CI 11.7–54.6) |
| Hypoglycaemia (glucose <40 mg/dL) | 0 (0.0%; 95% CI 0.0–5.4) | 0 (0.0%; 95% CI 0.0–7.0) | 0 (0.0%; 95% CI 0.0–21.5) |
| Metabolic acidosis (pH <7.25 or HCO₃⁻ <15 mmol/L) | 2 (3.0%; 95% CI 0.8–10.2) | 2 (3.9%; 95% CI 1.1–13.5) | 0 (0.0%; 95% CI 0.0–21.5) |
| Hyperlactataemia (lactate >5 mmol/L) | 1 (1.5%; 95% CI 0.3–8.0) | 1 (2.0%; 95% CI 0.3–10.3) | 0 (0.0%; 95% CI 0.0–21.5) |
| Haemoglobinuria (massive haemolysis) | 5 (7.5%; 95% CI 3.2–16.3) | 5 (9.8%; 95% CI 4.3–21.0) | 0 (0.0%; 95% CI 0.0–21.5) |
| Jaundice (indirect bilirubin >50 µmol/L or >3 mg/dL) associated with other severity signs | 11 (16.4%; 95% CI 9.4–27.1) | 9 (17.6%; 95% CI 9.7–30.2) | 2 (14.3%; 95% CI 4.0–39.9) |

*Values are presented as n (%) with Wilson 95% confidence intervals. Patients may have met more than one severity criterion; therefore, criterion counts do not sum to the number of patients with any clinical or biochemical criterion.*

*Hyperparasitaemia was not evaluated because parasitaemia was recorded using a semiquantitative cross-based microscopy system.*

*Abbreviations: ARDS, acute respiratory distress syndrome; CI, confidence interval; DIC, disseminated intravascular coagulation; GCS, Glasgow Coma Scale; HCO₃⁻, bicarbonate; SBP, systolic blood pressure.*

**Supplementary Table 4.** Laboratory parameters of the overall study population before and after treatment with at least one dose of intravenous artesunate.

| **Laboratory Parameter** | **Pre-treatment, median (IQR)** | **Post-treatment, median (IQR)** | **p-value** |
| --- | --- | --- | --- |
| Haemoglobin (g/dL), median (IQR) | 10.97 (9.07–12.97) | 9.99 (8.11–11.60) | 0.0045 |
| Haematocrit (%), median (IQR) | 32.70 (27.40–38.30) | 30.0 (25.45–34.75) | 0.0089 |
| Leukocytes (×10³/µL), median (IQR) | 6.5 (4.50–8.50) | 5.82 (4.28–7.42) | 0.0472 |
| Platelets (×10³/µL), median (IQR) | 69.10 (36.95–117.50) | 114.3 (62.50–206.70) | <0.0001 |
| Creatinine (mg/dL), median (IQR) | 0.75 (0.59–0.95) | 0.65 (0.55–0.83) | ns* |
| Glucose (mg/dL), median (IQR) | 106.0 (90.60–125.0) | 97.15 (86.71–115.2) | 0.05 |

*Values are presented as median (interquartile range).*

** ns = non-significant.*

*Abbreviations: IQR, interquartile range; ns, non-significant.*

**Supplementary Table 5.** Laboratory parameters among patients with severe malaria, according to infecting Plasmodium species, before treatment and after at least one dose of intravenous artesunate.

| **Laboratory parameter** | ***P. vivax* (n=51); median (IQR)** | ***P. falciparum* (n=14); median (IQR)** | **p value** |
| --- | --- | --- | --- |
| **Laboratory parameters – Severe malaria (baseline)** | | | |
| Haemoglobin (g/dL) | 10.80 (8.20–12.59) | 10.30 (5.90–12.02) | 0.228 |
| Haematocrit (%) | 32.05 (26.95–36.48) | 31.50 (19.90–34.70) | 0.530 |
| Leukocytes (×10³/µL) | 6.53 (5.30–8.46) | 7.39 (5.19–7.71) | 0.937 |
| Platelets (×10³/µL) | 50.0 (33.0–103.8) | 131.0 (97.0–209.0) | 0.0020 |
| Creatinine (mg/dL) | 0.80 (0.63–1.09) | 0.63 (0.52–0.91) | 0.060 |
| Glucose (mg/dL) | 113.1 (92.5–130.0) | 91.42 (86.29–107.7) | 0.0279 |
| **Laboratory parameters – Severe malaria (after ≥1 dose of intravenous artesunate)** | | | |
| Haemoglobin (g/dL) | 9.48 (7.43–11.12) | 9.45 (8.42–10.06) | 0.7864 |
| Haematocrit (%) | 29.20 (24.70–33.70) | 29.50 (27.15–31.00) | 0.7576 |
| Leukocytes (×10³/µL) | 5.98 (4.20–7.28) | 6.69 (3.38–8.41) | 0.7221 |
| Platelets (×10³/µL) | 87.15 (55.98–161.3) | 168.50 (74.75–255.0) | 0.1049 |
| Creatinine (mg/dL) | 0.65 (0.58–0.85) | 0.53 (0.47–0.70) | 0.2168 |
| Glucose (mg/dL) | 98.88 (90.58–119.6) | 90.50 (73.83–108.5) | 0.0568 |

*Values are presented as median (interquartile range).*

*Abbreviations: IQR, interquartile range.*

**Supplementary Table 6.** Laboratory parameters among patients with non-severe malaria, according to infecting Plasmodium species, before treatment and after at least one dose of intravenous artesunate.

| **Laboratory parameter** | ***P. vivax* (n=46); median (IQR)** | ***P. falciparum* (n=13); median (IQR)** | **p value** |
| --- | --- | --- | --- |
| **Laboratory parameters – Non-severe malaria (baseline)** | | | |
| Haemoglobin (g/dL) | 12.30 (10.20–13.70) | 10.10 (8.46–11.15) | 0.0128 |
| Haematocrit (%) | 36.65 (30.20–40.0) | 30.05 (25.50–33.33) | 0.0042 |
| Leukocytes (×10³/µL) | 6.74 (4.18–8.89) | 5.56 (3.71–8.33) | 0.289 |
| Platelets (×10³/µL) | 68.0 (32.9–90.95) | 117.2 (57.25–244.8) | 0.0281 |
| Creatinine (mg/dL) | 0.76 (0.59–1.0) | 0.47 (0.42–0.51) | 0.0001 |
| Glucose (mg/dL) | 108.7 (94.85–130.8) | 86.11 (66.71–109.5) | 0.0060 |
| **Laboratory parameters – Non-severe malaria (after ≥1 dose of intravenous artesunate)** | | | |
| Haemoglobin (g/dL) | 11.0 (8.37–12.65) | 9.25 (7.69–10.86) | 0.1752 |
| Haematocrit (%) | 32.80 (25.33–36.80) | 29.45 (23.95–32.78) | 0.2244 |
| Leukocytes (×10³/µL) | 5.63 (4.69–7.26) | 6.29 (3.77–7.18) | 0.8054 |
| Platelets (×10³/µL) | 114.0 (65.15–189.2) | 147.0 (120.3–331.8) | 0.0532 |
| Creatinine (mg/dL) | 0.71 (0.58–0.84) | 0.45 (0.40–0.48) | 0.0680 |
| Glucose (mg/dL) | 100.6 (88.16–115.3) | 86.83 (60.64–98.75) | 0.0005 |

*Values are presented as median (interquartile range).*

*Abbreviations: IQR, interquartile range.*
